# Single loading-dose tafenoquine for malaria chemoprophylaxis during brief travel?

**DOI:** 10.1093/jtm/taab081

**Published:** 2021-05-28

**Authors:** J Kevin Baird

**Affiliations:** Eijkman-Oxford Clinical Research Unit, Eijkman Institute of Molecular Biology, Jakarta, Indonesia; Centre for Tropical Medicine and Global Health, Nuffield Department of Medicine, University of Oxford, Oxford, UK

**Keywords:** Malaria, Chemoprophylaxis, Tafenoquine, Single dose, Brief travel

In this issue of the *Journal of Travel Medicine*, Islam *et al.*^1^ examine what the US Army developers of tafenoquine envisioned as its most useful application—a single dose providing sustained protection from malaria. Informally, they referred to that vision as ‘fire-and-forget’ chemoprophylaxis. The relatively prolonged elimination half-life of tafenoquine (about 18 days) was selected from among competing preclinical 8-aminoquinolines for this purpose.^2^ However, none of the trials supporting the registration of tafenoquine for chemoprophylaxis (by the US FDA in 2018) assessed single dose efficacy during brief travel. Walsh *et al.*^3^ demonstrated 400-mg monthly tafenoquine dosing provided good efficacy in Thai soldiers over 5 months. The other trials involved 200-mg weekly prophylaxis of Australian soldiers on prolonged travel (6mo) or permanent residents of endemic areas as subjects.^3–7^ A trial involving sufficient numbers of subjects exposed to relatively brief and sufficiently high-risk of infection to definitively demonstrate single-dose, short-term protection was not done. It may be improbable that such a trial could be done outside of the laboratory because such populations very rarely occur. The report from Islam *et al.*^1^ approaches that ideal by a pragmatic analysis of existing trial data.

Nine randomized controlled trials of tafenoquine chemoprophylaxis involving 1714 subjects followed for *<*28 days (short-term) or >28 days (long-term) post-dosing were assessed.^1^ Those follow-up intervals served as hypothetical periods of travel. Subjects received either a single loading dose of 600-mg tafenoquine (L-TQ; daily 200 mg × 3 d), loading dose plus weekly 200-mg tafenoquine (TQ), weekly mefloquine (MQ) or a placebo. Whereas TQ and L-TQ each showed equal efficacy to MQ short-term, only TQ was equal to MQ long-term. The results indicate that a single pre-travel loading dose of 600 mg (over 3 days) tafenoquine may protect against parasitemia as well as weekly mefloquine or tafenoquine during travel of less than a month. We cannot know if protection against later relapses occurred, but that may be at least provisionally presumed for this drug.

Tafenoquine is probably causally protective rather than suppressive. That is, it prevents the formation of active and latent forms of plasmodia in the liver rather than killing them after they emerge into the bloodstream. This provides the tremendous advantage of also protecting against latent *Plasmodium vivax* and *Plasmodium ovale* malarias. No other chemoprophylactic antimalarials do so, excepting only daily primaquine prophylaxis.^8^^,^^9^ This quality spares travellers the requirement of post-travel dosing, be it with presumptive anti-relapse therapy (PART) with primaquine (14 days) or continued suppressive prophylaxis (1–4 weeks). Absent those measures, clinical attacks often occur in the weeks and months following travel under suppressive prophylaxis.^10^ Tafenoquine and primaquine are 8-aminoquinolines and each provokes acute hemolytic anemia in patients having inherited glucose-6-phosphate dehydrogenase (G6PD) deficiency,^11^ imposing the necessity of screening prior to dosing.^12^

Conventional suppressive prophylaxis has never been well suited to brief travel at high risk of malaria. Weeks of dosing for days of protection adds up to great inconvenience, improbable acceptance and poor adherence. Daily primaquine chemoprophylaxis has been considered a good option for brief travel and to where *P. vivax* is the dominant risk.^13^^,^^14^ The traveller may commence dosing hours prior to travel and cease dosing a day or two following travel with assurance of good efficacy while traveling, and without concern for post-travel clinical attacks due to latency.

The development of primaquine during the 1940s and 1950s employed sporozoite challenge of prisoner volunteers; a model that allowed ascertaining that a single 30-mg dose of primaquine administered within 2 days of inoculation (but not 15 mg, or 30 mg just a day later) effectively prevented both *Plasmodium falciparum* and *P. vivax* malarias.^8^ Early hepatic plasmodia are vulnerable to relatively very low doses of primaquine whereas more mature forms are not; i.e. 30 mg for early forms vs. 210–420 mg for more mature forms in the instance of *P. vivax* hypnozoites. This definitive evidence underpins the use of primaquine for causal chemoprophylaxis. As already explained, the developers of tafenoquine employed no such clinical model and its chemoprophylactic properties are much less well understood.

The practice of load dosing at the front of chemoprophylaxis stems from decades of conventional suppressive chemoprophylaxis practice. That served the purpose of bringing plasma levels quickly up to those compatible with suppressing asexual parasitemia. That practice is unnecessary in causal prophylaxis with primaquine—the lone daily dose within 48 h of sporozoite inoculation is all that is required. The developers of tafenoquine included the loading dose practice because they did not know, and still do not, if its chemoprophylactic protection is purely causal. We may reasonably consider the possibility that a single dose of tafenoquine immediately prior to brief travel may suffice.

This is perhaps the key question with tafenoquine prophylaxis: what single dose kills the early hepatic forms of plasmodia for how long? The 200 mg daily for 3 days pre-travel dose of tafenoquine may be in excess of what causal prophylaxis requires for a month of protection, and in great excess of protection for a few days or weeks of travel. Although the efficacy of L-TQ waned beyond 28 days in the study of Islam *et al.*,^1^ very substantial protection nonetheless persisted; the odds ratio of parasitemia relative to MQ was 2.9 for L-TQ, whereas with placebo, it was 62.9. In the tafenoquine prophylaxis dose-ranging trial of Hale *et al.*,^6^ just 50-mg loading (daily for 3 days) followed by weekly 50-mg tafenoquine was 84% efficacious against *P. falciparum* in Ghanaian adults over 13 weeks exposure to intense transmission—the 200-mg dose was 86% efficacious (both doses being equivalent to the mefloquine comparator). A loading dose of tafenoquine well below the 600-mg standard seems highly likely to be effective for travel of short duration, i.e. days to a few weeks.

This is an important question taken in light of the problem of G6PD deficiency with tafenoquine. Whereas a 200-mg single dose caused the hematocrits 3 of 6 healthy G6PD heterozygotes to drop by more the 7%, none of 6 subjects dosed with 100 mg did so.^11^ The planned escalation of that dose to 600 mg in that trial did not occur because the hemolytic reactions to the 300-mg dose halted escalation. A single dose of tafenoquine well below 100 mg may conceivably be safely administered to patients without G6PD screening.

For the time being, the minimal dose and duration of causal efficacy with single-dose tafenoquine without loading remains wholly unknown. A minimal dose calibrated to brief durations of exposure, and perhaps tolerable to G6PD-deficient patients, may plausibly be learned. Greater assurance and dosing precision could come from experimental sporozoite challenge of macaques with *Plasmodium cynomolgi* or of humans with *P. vivax* administered a range of single doses of tafenoquine days prior to single or repeated challenge at intervals revealing the duration of causal prophylaxis. Ascertaining those values may enable convenient, safe and effective prevention of malaria in travellers making brief visits to high risk areas.

Malaria chemoprophylaxis—long seen as primarily benefitting well-heeled international adventure seekers or armies—may become an important instrument of public health for nations nearing malaria elimination goals. Domestic travel from areas of no-risk to high-risk and back poses a threat both to the traveller and his or her community. Interrupted malaria transmission rarely means elimination of local anopheline vector mosquitoes, and resumption of transmission requires only a single infectious human carrier. Progression from case to outbreak to epidemic, and finally to sustained endemic transmission may occur within just a few weeks or months (erasing many years of hard work). Protecting malaria elimination achievements may be more likely to succeed with protecting residents venturing into relatively nearby areas of active transmission. In a global health sense, the military vision of highly convenient and effective fire-and-forget causal prophylaxis may be of great utility in the very difficult task of achieving and sustaining malaria elimination across subnational landscapes of shifting and patchy transmission. Discovery of the minimal dose and duration of single-dose tafenoquine chemoprophylaxis may well realize that vision.
